# Unilateral Muscle Overuse Causes Bilateral Changes in Muscle Fiber Composition and Vascular Supply

**DOI:** 10.1371/journal.pone.0116455

**Published:** 2014-12-29

**Authors:** Yafeng Song, Sture Forsgren, Jing-Xia Liu, Ji-Guo Yu, Per Stål

**Affiliations:** 1 Department of Integrative Medical Biology, Section for Anatomy, Umeå University, Umeå, Sweden; 2 Department of Surgical and Perioperative Sciences, Sports Medicine Unit, Umeå University, Umeå, Sweden; West Virginia University School of Medicine, United States of America

## Abstract

Unilateral strength training can cause cross-transfer strength effects to the homologous contralateral muscles. However, the impact of the cross-over effects on the muscle tissue is unclear. To test the hypothesis that unilateral muscle overuse causes bilateral alterations in muscle fiber composition and vascular supply, we have used an experimental rabbit model with unilateral unloaded overstrain exercise via electrical muscle stimulation (E/EMS). The soleus (SOL) and gastrocnemius (GA) muscles of both exercised (E) and contralateral non-exercised (NE) legs (n = 24) were morphologically analyzed after 1w, 3w and 6w of EMS. Non-exercised rabbits served as controls (n = 6). After unilateral intervention the muscles of both E and NE legs showed myositis and structural and molecular tissue changes that to various degrees mirrored each other. The fiber area was bilaterally smaller than in controls after 3w of E/EMS in both SOL (E 4420 and NE 4333 µm^2^ vs. 5183 µm^2^, p<0.05) and GA (E 3572 and NE 2983 µm^2^ vs. 4697 µm^2^, p<0.02) muscles. After 6w of E/EMS, the percentage of slow MyHCI fibers was lower than in controls in the NE legs of SOL (88.1% vs. 98.1%, p<0.009), while the percentage of fast MyHCIIa fibers was higher in the NE legs of GA (25.7% vs. 15.8%, p = 0.02). The number of capillaries around fibers in the E and NE legs was lower (SOL 13% and 15%, respectively, GA 25% and 23%, respectively, p<0.05) than in controls. The overall alterations were more marked in the fast GA muscle than in the slow SOL muscle, which on the other hand showed more histopathological muscle changes. We conclude that unilateral repetitive unloaded overuse exercise via EMS causes myositis and muscle changes in fiber type proportions, fiber area and fiber capillarization not only in the exercised leg, but also in the homologous muscles in the non-exercised leg.

## Introduction

It is widely accepted that unilateral strength training increases strength not only in the trained muscle but also to some extent in the homologues muscle of the contralateral limb [1]–[4]. This effect has been reported for both small and large limb muscles and can occur by various modalities of exercise (E) that is accomplished by voluntary efforts as well as by electrical muscle stimulation (EMS) [5]–[10]. The potential of the effect to induce strength enhancement in the untrained contralateral muscle is of great interest and has obvious relevance in clinical rehabilitation. Unilateral exercise has for example been reported to prevent contralateral immobilized limbs in healthy individuals from loss of muscle mass and strength [11]. Even a bout of eccentric exercise may have a protective effect in the contralateral limb [12], [13]. It has also been demonstrated that exercise induced via EMS resulted in greater contralateral effects than that induced by voluntary contraction training [4], [9], [14]. However, adverse events may also be cross-transferred. Recently we reported that unilateral muscle overuse by E/EMS leading to injury in the rabbit triceps surae muscle also affected the homologous contralateral muscle. In that study we found focal histopathological muscle changes and inflammation (myositis) in both the manipulated and the resting leg [15]. Furthermore, there was an bilateral up-regulation of neuropeptide substance P, a neuromodulator in the tachykinin family that is known to be involved in neurogenic inflammation and vasodilatation [16]. Contralateral changes in the peripheral nerve system following unilateral nerve injury have been reported in numerous studies (for review see [17]), findings supporting the idea that deleterious events in a muscle can be cross-transferred. Further support for a cross-transfer activity of the nervous system is the often strictly symmetrical distribution of rheumatoid arthritis and some chronic inflammatory disease and a mirror image of the nervous system for pain [18]–[21]. Although cross-over effects are well described in the literature, it remains unclear whether unilateral overuse exercise cause structural or molecular changes in the contralateral muscles.

Skeletal muscles have an adaptive potential to modify their composition of muscle phenotypes and their metabolic profile in response to altered patterns of activity [22]–[24]. This adaptive response reflects, among several other factors, the ability of a muscle cell to modulate the type of contractile myosin heavy chain (MyHC) isoforms, size of the fibers [25], mitochondrial oxidative capacity and capillary supply of the muscle cells [26]–[29]. To our knowledge, no study has systematically examined alterations in the expression of major contractile MyHC proteins, muscle fiber size and capillary supply when the contralateral muscles has been influenced by exercise via EMS.

In the present study, a rabbit exercise model was used to test the hypothesis that unilateral unloaded overstrain induced by E/EMS not only causes bilateral histopathological tissue alterations and inflammation [15], [16], but also bilateral changes in the contractile motor system and in the vascular compartment. The triceps surae muscle of one leg was exposed to repeated E/EMS for three experimental periods, 1, 3 and 6 weeks. After each experimental period both the exercised and non-exercised muscles were analyzed with enzyme and immunohistochemical and morphological techniques for determining possible changes in fiber phenotype compositions, fiber morphology and capillary supply. The advantage of using the triceps surae muscle is that its two major muscle parts, the soleus and gastrocnemius muscles, in various species substantially differ from each other in muscle fiber type composition [30], [31].

## Materials and Methods

### Ethics statement

The study was approved by the ethical committee at Umeå University (protocol A34/07) that complied with national (SFS1988:534; 1988:539) and international (2010/63/EU) guidelines and standards in animal research. The approval was obtained before the start of the study. A licensed breeder had bred all the animals for the sole purpose of being used in animal experiments. All efforts were made to minimize animal suffering.

### Animals

The experiment was carried out on female New Zealand white rabbits aged from 6 to 9 months and weighing approximately 4 kg. In total, 24 rabbits were included in the experiment, of which 18 were distributed in three experiment groups and the others served as controls (6 rabbits in each group). Throughout the experiment, the rabbits were kept under anaesthesia, induced by intramuscular injections of diazepham (0.2 ml/kg) and fentanylfluanisone (0.2–0.3 ml/kg). To maintain anaesthesia, fentanylfluanison (0.1 ml/kg) was further injected every 30–45 min during the experiment procedure. Buprenorphine (0.01–0.05 mg/kg) was given s.c. postoperatively to minimize pain. The six animals constituting the control group were kept in ordinary cages but had not been subjected to the exercise regimen in the experiment.

### Experimental design

An experimental model using a kicking machine, constructed for unloaded exercise via simultaneous electrical muscle stimulation of three rabbits, was used for the achievement of passive flexions and extensions of the right ankle joint. This model is a modified form of the model that was used by Backman et al. [32], [33], and that has previously been used to examine tendon changes (tendinopathy/tendinosis) in the Achilles tendon [32], [33]. A pneumatic piston attached to the right foot produced the movements of the ankle joint, in which the range of motion could be controlled. The range of movement was set to 9.5 cm, given a range of motion in the ankle of 55–65°, of which 20–25° was dorsiflexion and 35–40° was plantar flexion. A band was tied around the pelvis/hip of the rabbit to restrict movement in the left non-exercised leg. During the plantar flexion, an active concentric contraction was induced by electrical stimulation via surface electrodes (pediatric electrode 40 426A, Helwett Packard, Andover, MA, USA), placed 2 cm apart over the triceps surae muscle. The stimulation was synchronized with the plantar flexion movement of the piston by a microswitch, which trigged the stimulator unit (Disa stimulator Type 14E 10; Disa Elektronik A/S, Herlev, Denmark). The stimulator generated an impulse with a duration of 0.2 ms that was delivered 85 ms after the initiation of the plantar flexion at amplitude of 35 to 50 V. The stimulus was controlled by an oscillometer and the frequency of the flexion and extension movements was set to 150 per minute (2.5 Hz) and the repetitive movement was set to a period of 2 hours. The experiment was repeated every second day and the length of the experimental periods was 1 week, 3 weeks and 6 weeks. The rabbits were kept in ordinary cages allowing freedom of movement in-between the experimental periods. After each experimental period, the animals were sacrificed (the day after the last experiment session) by intravenous overdose of Pentobarbital. Thus, animals examined after an experimental period of 1 week had been subjected to the experimental sessions on four occasions. The control animals were sacrificed in connection to the start of the experiments. For further details, see Backman et al. [32], Andersson et al. [33] and Song et al. [15].

### Muscle samples

After the animals were sacrificed, the triceps surae muscle was dissected out from both experimental and contralateral sides. The soleus and the gastrocnemius muscles of the triceps surae were identified and muscle samples of an approximate size of 5×5–10 mm were obtained from the same area in the distal portions of the muscles of both legs. In the gastrocnemius muscle the sample were obtained from the central/distal part of the muscle, while the sample from the soleus muscle represented the entire cross-section of the muscle belly. The muscle specimens were directly mounted in OCT compound (Tissue Tek, Miles laboratories, Naperville, IL, USA) on a thin cardboard and rapidly frozen in propane chilled with liquid nitrogen, and stored at −80°until use. Five and 8 µm thick serial cross-sections were cut in a cryostat microtome (Rechert-Jung, Leica Heidelberg, Germany) at −23°C and mounted on glass slides.

### Immunohistochemistry

The 5 µm thick muscle cross-sections were processed for immunhistochemistry (IHC) with previously characterized monoclonal antibodies (mAbs) and one polyclonal antibody (Ab). The major contractile proteins in muscle fibers, myosin heavy chain (MyHC) isoforms, were analysed by using different mAbs against developmental and adult MyHC isoforms (for antibody details and origin see Table 1) [34]–[37]. Visualization of muscle fiber contour (i.e. basal lamina) of muscle fibers was performed with staining for polyclonal Ab Pc128 against laminin (Binding Site Group, UK, diluted 1∶15000) and mAb NCL-merosin against laminin α2-chain (Novocastra Laboratories Ltd, UK, diluted 1∶1000) [38]. MAb D33 (Dako, Denmark, diluted 1∶1000) against desmin was used for the detection of muscle fiber degeneration (increased staining) and fiber necrosis (no staining) and mAb 1891 against fibronectin (Chemicon, Temecula, CA, USA, diluted 1∶2000) was used for analysis of fibrosis. To identify capillaries, mAb M0823 against CD31 (Dako Glostrup, Denmark, diluted 1∶100) that recognizes PECAM-1 (platelet endothelial cell adhesion molecule), a transmembranous glycoprotein strongly expressed by vascular endothelial cells, was used. MAbs against CD31 has previously been shown to identify vascular endothelium in normal muscle [39], [40] and in rabbit tendons [33]. Axons in nerves were stained with mAb T8660 against β-III Tubulin (Sigma-Aldrich, USA, diluted 1∶500) and Schwann cells were stained with mAb S2532 against S-100beta (Sigma-Aldrich, USA, diluted 1∶500) [15], [41], [42].

**Table 1:** Data on primary antibodies used for detection of different MyHC isoforms.

Antibody	Specificity	Gene*	Reference
A4.451	MyHCI	MYH7	[34]
A4.74	MyHCIIa	MYH2	[35], [36]
N2.261	MyHCI	MYH	[35], [36]
	MyHCIIa	MYH2	
	MyHCeom	MYH13	
	MyHCα-cardiac	MYH6	
F1.652	MyHCembryonic	MYH3	[37]

All monoclonal Abs were obtained from The Developmental Studies Hybridoma Bank, developed under the auspices of the NICHD and maintained by The University of Iowa, Department of Biological Sciences, Iowa City, IA, USA.

*Official gene nomenclature according to OMIM (http://www.ncbi.nlm.nih.gov/omim/).

Immunostaining for all mAbs were performed using standard techniques. The sections were first rinsed in phosphate-buffered saline (PBS) for 3×5 min and then incubated for 20 min in a 1% solution of detergent Triton X-100 (Kebo Lab, Stockholm, Sweden) in 0.01M PBS, pH 7.2, containing 0.1% sodium azide. After this procedure the sections were rinsed 3×5 min in PBS and incubated in 5% normal rabbit serum in PBS supplemented with 0.1% bovine serum albumin (BSA) for 15 min in room temperature. All sections were thereafter incubated with the primary antibody diluted to appropriate concentrations in PBS with BSA in humid environment. Incubation proceeded for 60 min at 37°C. Double staining of the muscle cross-sections was performed with mAb M0823 (CD31) and polyclonal Ab Pc128 against laminin or with mAb NCL-merosin against laminin α2 and each of the four different mAbs against adult and developmental MyHCs (Table 1). After washes in PBS (3×5 min) and another incubation in normal rabbit serum, all sections except those double stained against merosin and MyHC isoforms (cf. below), were incubated with a secondary polyclonal rabbit anti-mouse TRITC (R0276, Dako, Denmark) diluted 1∶40 or Alexa Fluro 488 (A11029, Invitrogen, Carlsbad, CA, USA), diluted 1∶100 for 30 min at 37°C. The sections were thereafter washed in PBS 3×5 min and then mounted in Vectashield Mounting Medium (H-1000) or Mounting Medium with DAPI (H-1500) (Vector Laboratories, Burlingame, CA, USA) in order to identify nuclei. For further details of the laboratory procedures, see Song et al. [15] and Spang et al. [43].

Indirect peroxidase-anti-peroxidase (PAP) technique [44] was performed to visualize the double stained sections with mAbs against laminin α2 (mAb NCL-merosin) and the reactions for the different MyHC isoforms (Table 1). After incubation with the primary Abs, the sections were washed in 0.01M PBS for 15 min, incubated with 1% mouse peroxidase-antiperoxidase (Dakopatts, Glostrup, Denmark) for 30 min and then washed again in 0.01M PBS for 15 min. The peroxidase binding was revealed by applying a solution containing diaminobenzidine and hydrogen peroxidase (H2O2) for 10 min. Finally, the sections were rinsed with running water for 5 min, dehydrated in graded concentration of ethanol, followed by xylene treatment and mounting with DPX (BDH, Limited Poole, England).

For control of unspecific staining, sections were treated as described above, except that normal serum was used instead of primary antibodies. No specific staining was observed in these control sections. For further details of the staining procedures see Song et al. [15], Liu et al. [35], Spang et al. [43], Stål and Lindman [45] and Österlund et al. [46].

### Enzyme-histochemistry and basic histology

Muscle cross-sections, 8 µm thick, serial to those used for immunohistochemistry, were stained with Hematoxylin-eosin (HTX-eosin) for demonstration of basic histology, including detection of degenerative and regenerative processes and inflammation [47]. To distinguish classical fiber types by enzyme histochemistry, serial sections were stained for myofibrillar ATPase (mATPase) (EC 3.6.1.3) after preincubations at pH 10.3, 9.4, 4.6 and 4.3 [47].

### Muscle fiber classification

Based on the IHC staining pattern for the different MyHC mAbs, the fibers were classified as fibers containing pure MyHCI, MyHCIIa and MyHCIIx/b isoforms (since we could not distinguish between MyHCIIx and MyHCIIb with the used mAbs, fibers containing these MyHC isoforms were grouped together and classified as one fiber type) (Table 2). Fibers containing two or more MyHC isoforms were classified as hybrid fibers co-expressing MyHCI and MyHCIIa (MyHCI+IIa) or MyHCIIa and MyHC IIx/b (MyHCIIa+IIx/b). The fiber type classification based on MyHC isoforms was further controlled with classical fiber type classification based on myofibrillar ATPase (mATPase) staining pattern after alkaline and acid preincubations (c.f above). For detailed description of mATPase fiber type classification see [47].

**Table 2:** The basis for the classification of muscle fibre phenotypes based on the staining intensity pattern of the different MyHC mAbs.

mAb	MyHCI	MyHCI+MyHCIIa	MyHCIIa	MyHCIIa+MyHCIIx/b	MyHCIIx/b
N2.261	+, ++	+, ++	+++	+, ++	−
A4.451	+++	+, ++	−	−	−
A4.74	−	+, ++	+++	+, ++	−

(+) weak staining, (++) moderate staining, (+++) strong staining.

### Morphometric analyses

Three to six randomly chosen areas from each muscle sample were scanned in a light microscope (Zeiss Axiophot, Carl Zeiss, Oberkochen, Germany) equipped with an MTI CCD 72 video camera (DAGE-MTI, Michigan City, USA). Morphometric analysis of the scanned muscle areas was performed with an image analysis program (Image-Pro Plus, Media Cybernetics, Silver Spring, MD). Each muscle fiber in the chosen areas was classified into fiber types based on the expression of MyHC isoforms in the fibers (c.f. above). To estimate muscle fiber cross-sectional area (CSA) and number of capillaries around fibers, the circumference of each fiber and the associated capillaries were traced along the periphery of the basement membrane on a computer image. Muscle fibers and capillaries in the areas with myositis and fibrosis (c.f. below) were not included in the measurements as they could highly bias the calculation. In total, 11 676 fibers were included for the calculation of fiber area and capillary fiber supply (average 139 fibers per section, range 124–197). A total of 70 056 capillaries were included in the calculation of capillary density. Here it should be recalled that fifty fibers have previously been shown to be an adequate number for making a robust analysis of the capillary supply of muscle fibers in normal limb muscles [48].

### Capillary variables

All capillaries in contact, or nearly in contact, with individual muscle fibers were included in the analysis of number of capillaries around fibers (CAF). Capillaries related to each fiber relative to their fiber cross-sectional area (CAFA) were calculated according to the formula; CAF/fiber cross-sectional area ×10^3^. This variable relates the number of capillaries around fibers to fiber size and measures the cell area that each capillary supplies. Capillary density (CD) was calculated as the total number of capillaries per mm^2^ muscle cross-sectional area.

### Statistical analysis

Mean and standard deviation (SD) were calculated for descriptive statistics. A two-way analysis of variance test (ANOVA) was used for analysis of differences in mean value of each analyzed parameter in the three experimental groups and the controls. The analysis of normality in the distribution of the samples showed no indications of a skewed distribution within each group. All statistical analysis was performed with the statistical software SAS/STAT, 9.2 (SAS Institute Inc, USA). A p-value<0.05 was considered to be significant.

## Results

### General histopathology

**Figure 1:**
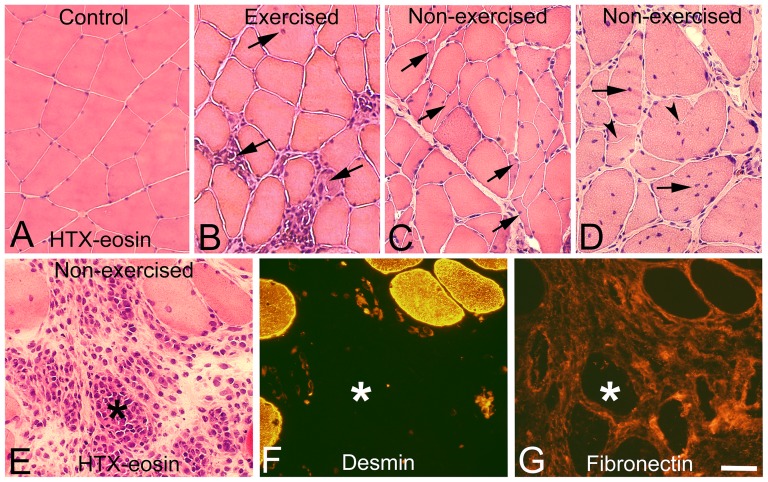
Focal muscle changes and myositis in the exercised and non-exercised leg. Muscle samples from a control (A), an exercised (B) and a contralateral non-exercised soleus (C–G) muscles after 6w of E/EMS. Sections A–E are stained with hematoxylin-eosin (HTX-eosin), section F is immunolabeled for mAb D33 against desmin and section G for mAb 1891 against fibronectin. Figs. E–G are serial sections. Note the infiltration of inflammatory cells (B, E), presence of internal nuclei (arrows B, D) and atypical formed fibers in both the exercised and non-exercised muscles (B–E). Small angular fibers are shown in Fig C (arrows). Presence of extremely large fiber size variability, split fibers (arrowheads) and increased number of internal nuclei (arrows) are shown in Fig D. A necrotic fiber (asterisk) within in an area with inflammation is shown in E–G. Note the severe infiltration of inflammatory cells in and around the necrotic fibers (E), the lack of desmin staining of the necrotic fibers (F) and the extensive fibrosis in the area with myositis and necrotic fibers (G). Scale bar 50 µm.

**Figure 2:**
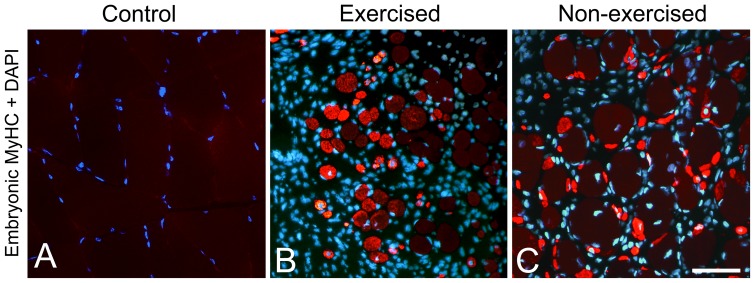
Bilateral regeneration of muscle fibers. Muscle cross-sections immunolabeled with mAb F1.652 against embryonic MyHC and DAPI (stains nuclei blue) in a control (A), an exercised (B) and a non-exercised (C) gastrocnemius muscles after 6w of E/EMS. Note the lack of staining for embryonic MyHC in the control sample (A) and the high number of small sized fibers stained for embryonic MyHC in areas with inflammation in the exercised and non-exercised muscles (B, D). Note also the higher variability in size as well as the generally larger sizes of fibers expressing embryonic MyHC in the exercised than in the non-exercised side, indicating that the degenerative/regenerative process is earlier initiated in the exercised than in the non-exercised side. Scale bar 50 µm.

**Figure 3:**
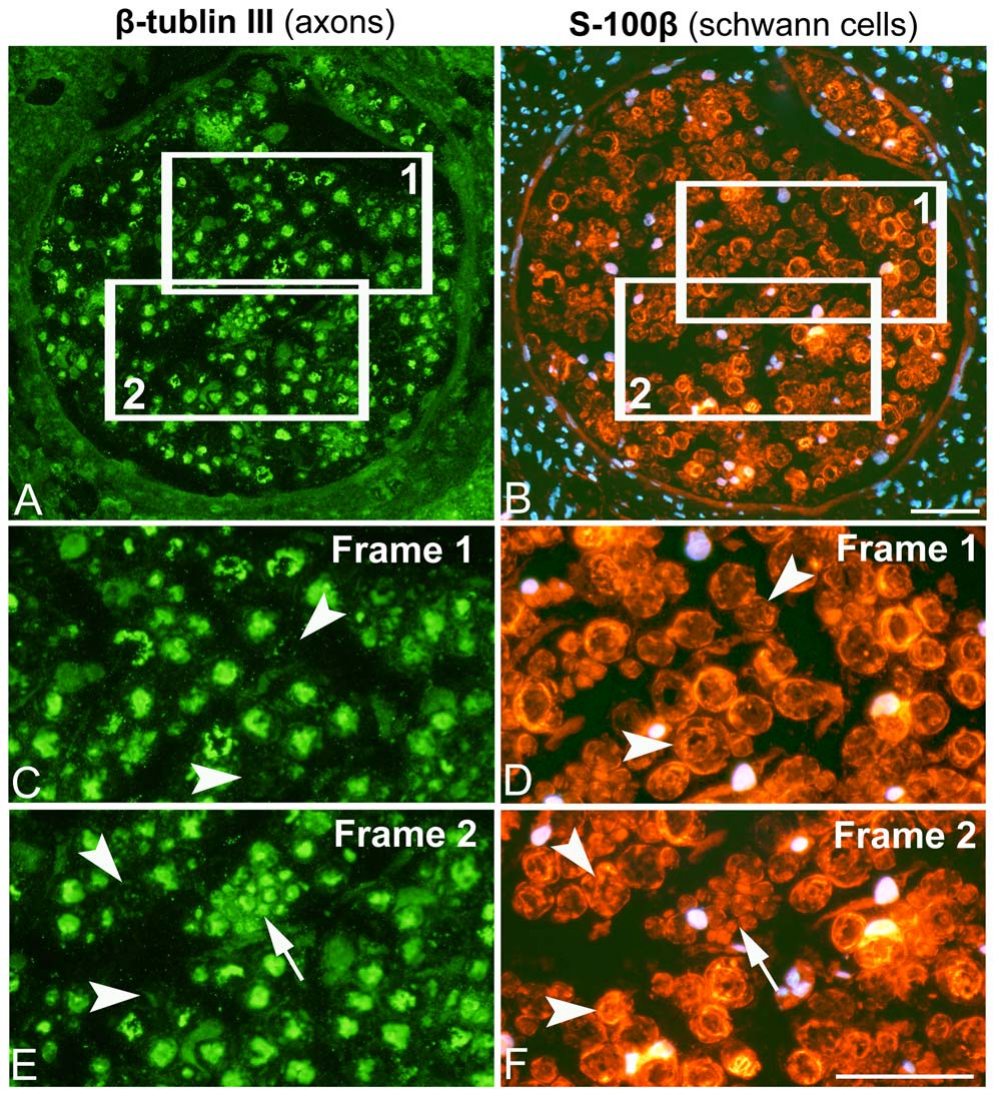
Changes in nerve fascicles in the non-exercised leg. Serial section of a nerve fascicle in the non-exercised soleus muscle after 6w of exercise immunolabeled for mAb β-tublin III against axons (A, C, E) and mAb S-100β against Schwann cells and DAPI (B, D, F). Nuclei are stained blue or pink with DAPI. The frames 1 and 2 inserted in figs. A and B corresponds to the regions shown in Figs. C, E and D, F, respectively. Axons stained for mAb β-tublin III are enclosed by Schwanns cells stained by mAb S-100β. Certain Schwann cell structures lack staining for mAb β-tublin III, indicating loss of axons (C–F, arrowheads). The group of small sized axons stained for β-tublin III in frame 2 (E,F, arrows) indicates on a process of regeneration by sprouting of axons (E, F). Scale bar 50 µm.

**Figure 4:**
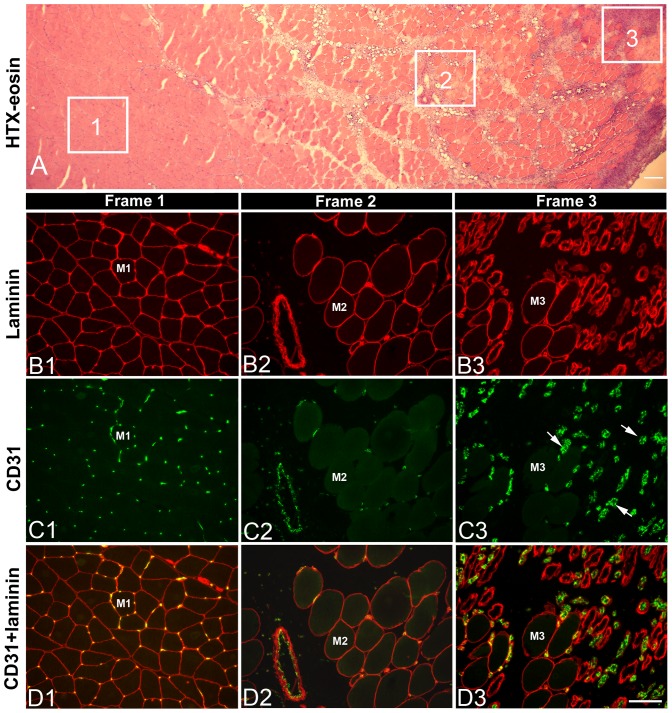
Blood vessel distribution in normal and pathological muscle areas. The figure shows an example of blood vessel distribution in areas with normal morphology and in areas with pathological changes in the exercised gastrocnemius muscle after 3w of E/EMS. Section A is stained with HTX-eosin and shows an area with normal morphology (1), an pathological area with fibrosis and fat infiltration (2) and an area with severe mysositis and muscle fiber alterations (3). Sections B1-B3 are immunolabeled for polyclonal Ab PC128 against laminin, a component of the basement membranes of muscle fibers and vessels, and section C1–C3 are immunolabeled for mAb M0823 (CD31) against endothelium in vessels. Sections D1–D3 are merged images of immunolabeling for CD31 and laminin. Note the normal pattern of capillaries around fibers in the unaffected area (frame 1), the low number of capillaries in the area with fibrosis (frame 2), and the high number of comparatively larger vessels (arrows) in the area marked with inflammation (frame 3). Indications M1, M2 and M3 represent a serially sectioned muscle fiber in each frame stained for laminin (B), CD31 (C) and both laminin and CD31 (D). Scale bar 100 µm.

**Figure 5:**
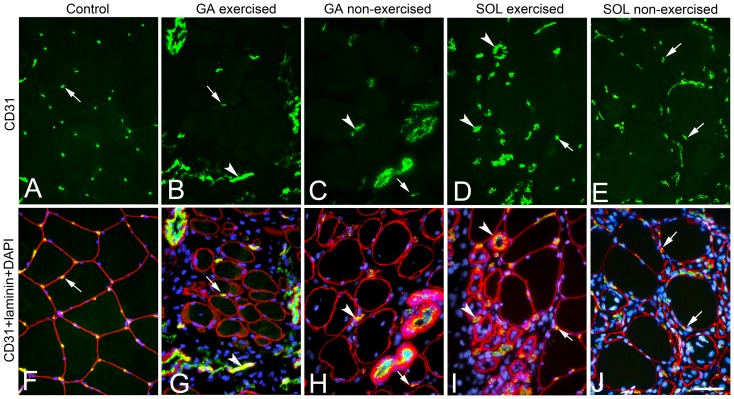
Bilateral changes in vascular supply in affected areas in the exercised and non-exercised gastrocnemius and soleus muscles. Serial cross-sections from a control (A, F), an exercised (B, G) and non-exercised (C, H) gastrocnemius (GA) muscle and an exercised (D, I) and non-exercised (D, H) soleus (SOL) muscle. The cross-sections A–E are stained for mAb M0823 (CD31) against endothelium in vessels and the merged sections E–J are stained for CD31 (yellow/green), pAb PC128 against laminin (red) and DAPI (stains nuclei blue). Sections A, F show a control with a normal pattern of capillaries around fibers whereas section B–E, F–J shows different patterns of changes in capillarization in areas with inflammation and fibrosis. The samples from the exercised and non-exercised GA muscles shows examples of low capillary density in an area with inflammation/fibrosis (B, G) and in an area with mainly fibrosis (C, H). The sample from the exercised soleus muscle (D, I) shows an example of increased number of vessels with a larger size than capillaries in an area with fibrosis, inflammation and degenerating/regenerating fibers. The sample from the non-exercised SOL (E, J) shows an area with severe inflammation and increased capillarization. The differences in vascularization patterns probably represent different stages in the degenerative/regenerative process in the muscles. Arrows point at immunoreactions for capillaries and arrowheads against larger vessels in each section. Scale bar 50 µm.

After 3w and 6w of E/EMS, inflammatory cell infiltration (myositis), fiber necrosis, fibrosis and changes in fiber morphology were observed in focal regions of both the exercised (E) and non-exercised (NE) soleus and gastrocnemius muscles (for details, see Song et al., [15]) (Fig. 1). Some of the areas affected by myositis in both the exercised and non-exercised legs contained clusters of fibers expressing embryonic MyHC (Fig. 2). The fiber size, as well as the variability in size, for fibers expressing embryonic MyHC were in generally larger in the exercised than in the non-exercised side. Within or adjacent to the myositis areas, certain nerve fascicles showed signs of axon loss and axon regeneration (Fig. 3). Moreover, muscle areas with extensive infiltration of inflammatory cells and severe histopathological tissue changes had mostly a high density of capillaries often with enlarged lumina and/or an increased number of small arterioles or venules. Adjacent areas with less or no inflammation, but with a high amount of connective tissue and fat infiltration, had a low capillary density (Figs. 4 and 5). The overall histopathological changes were generally more severe in the soleus than in the gastrocnemius muscle, although there was an inter-individual variability in the severity of abnormalities within both muscles.

### Muscle fiber composition and capillary supply

**Figure 6:**
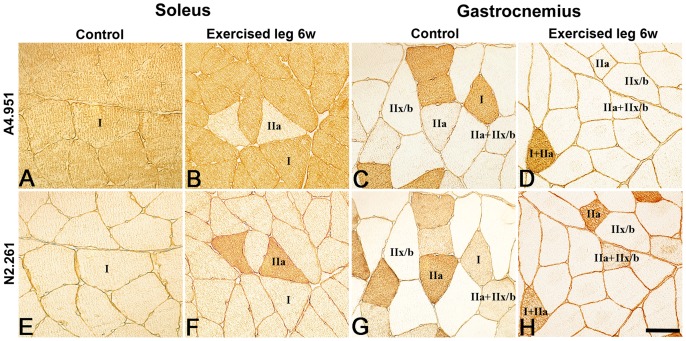
Muscle fiber phenotypes in the soleus and gastrocnemius muscles. Serial sections from the soleus (A, B and E, F) and gastronemius (C, D and G, H) muscles stained for mAb A4951 (MyHCI) (A–D) and mAb N2.261 (stains MyHCIIa strongly, MyHCI weakly) (E–H). Sections A, E and C, G are from controls and sections B, F and D, H are from the 6w exercised leg. Note the high frequency of fibers containing MyHCI in the soleus muscle (A, E) and the higher frequency of fibers containing MyHCIIx/b in the gastrocnemius muscle (C, G). Note also the higher number of fibers containing fast MyHCII in both muscles after 6w of E/EMS. Scale bar 50 µm.

**Figure 7:**
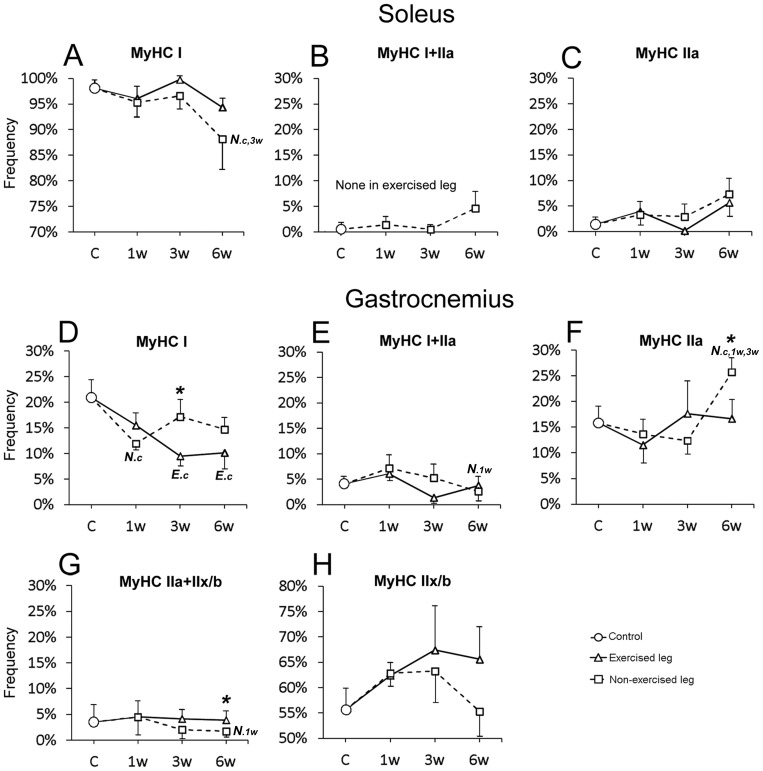
Graphs showing frequency of fiber types in the exercised and non-exercised muscles after 1w, 3w and 6w of unilateral exercise. Figs. A–C show the magnitude of changes in fiber type frequency (%) in the soleus muscles and Figs. D–H shows corresponding changes in the gastrocnemius muscles. The mean group values in the figure are connected with lines to facilitate interpretation of the direction of changes after E/EMS, but the lines do not imply that the changes between the sample points represent a linear change over this period. Exercised leg is defined by continuous black line, non-exercised leg by dotted line. Significant difference (p<0.05) within the exercised leg is marked with *E* and significant difference within the non-exercised leg is marked with *N*. Significant differences relative to controls are marked (c), to 1w group (1w) and to 3w group (3w). Significant differences (p<0.05) between exercised and non-exercised legs are marked with an asterisk (*). Note the shift against a lower proportion of fibers containing slow MyHCI and a higher proportion of fibers containing fast MyHCII in both exercised and non-exercised muscles. Note also the lack of hybrid MyHCI+IIa fibers in the exercised soleus muscle after exercise. Bars indicate SD.

**Figure 8:**
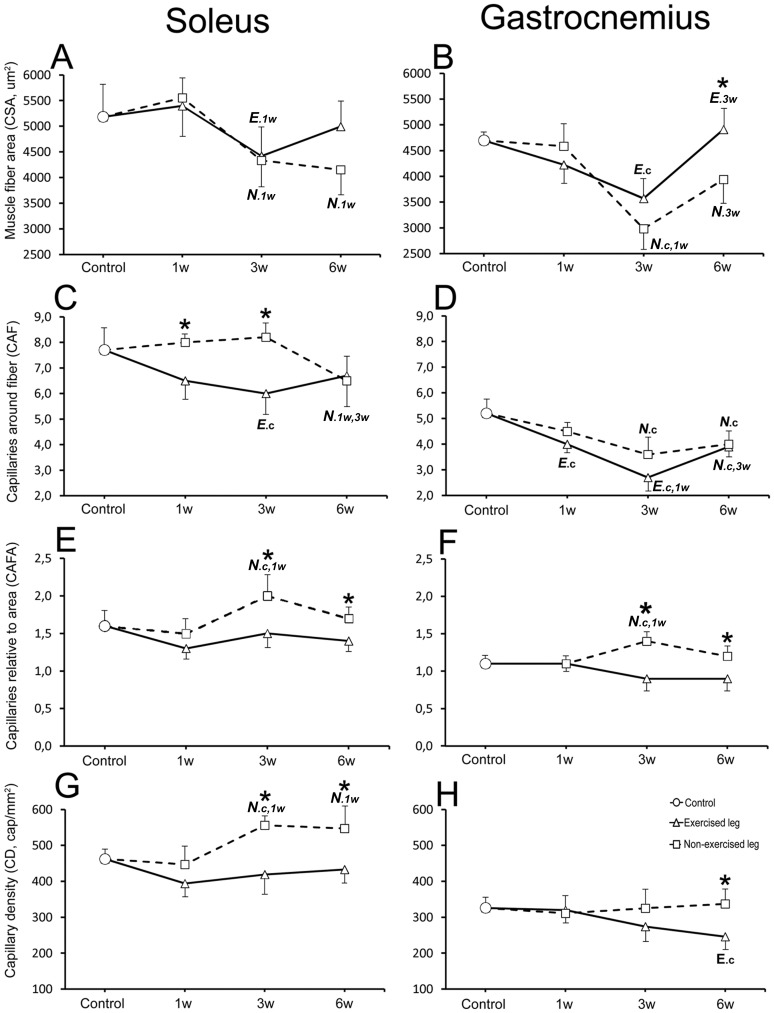
Graphs showing changes in fiber area and capillary parameters in the exercised and non-exercised muscles after 1w, 3w and 6w of unilateral exercise. Figures show changes in the exercised and non-exercised legs of soleus (A, C, E, G) and gastrocnemius (B, D, F, H) muscles for the following parameters; muscle fiber cross-sectional area (CSA) (A, B), the number of capillaries around each individual fiber (CAF) (C, D), the number of capillaries around fibers relative to cross-sectional fiber area (CAFA) (E, F), and capillary density (CD) (G, H). The mean group values in the figure are connected with lines to facilitate interpretation of the direction of changes after E/EMS, but the lines do not imply that the changes between the sample points represent a linear change over this period. Exercised leg is defined by continuous black line, non-exercised side by dotted line. Significant difference (p<0.05) within the exercised leg is marked with *E* and significant difference within the non-exercised leg is marked with *N*. Significant differences relative to controls are marked (c), to 1w group (1w) and to 3w group (3w). Significant differences (p<0.05) between exercised and non-exercised legs are marked with an asterisk (*). Note the overall similarities in changes in fiber area and in capillarization between the exercised and non-exercised legs in both the soleus and gastrocnemius muscles, except that there was no decrease in capillary supply after 1w and 3w of exercise in the soleus non-exercised muscle. Bars indicate SD.

**Figure 9:**
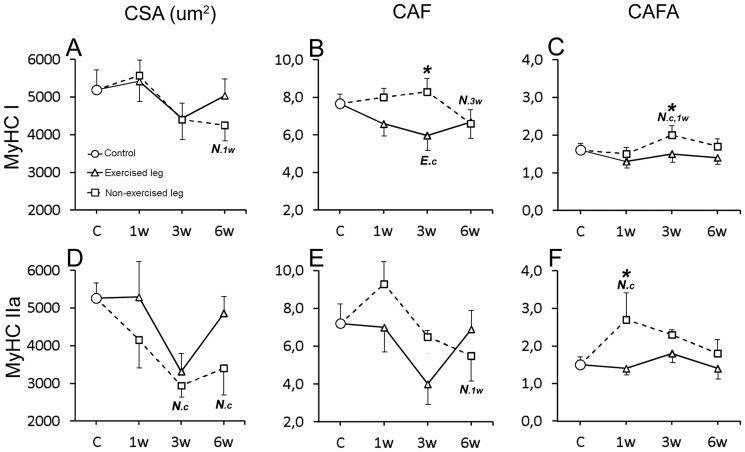
Graphs showing fiber area and capillary parameters for pure fiber types in the exercised and non-exercised soleus muscles after 1w, 3w and 6w of unilateral exercise. Figures show changes for the following parameters related to fibers containing MyHCI and MyHCIIa; muscle fiber cross-sectional area (CSA) (A, D), number of capillaries around each individual fiber (CAF) (B, E), and number of capillaries around fibers relative to cross-sectional fiber area (CAFA) (C, F). The mean group values in the figure are connected with lines to facilitate interpretation of the direction of changes after E/EMS, but the lines do not imply that the changes between the sample points represent a linear change over this period. Exercised side is defined by continuous black line, non-exercised side by dotted line. Significant difference (p<0.05) within the exercised leg is marked with ***E*** and significant difference within the non-exercised leg is marked with *N*. Significant differences relative to controls are marked (c), to 1w group (1w) and to 3w group (3w). Significant differences (p<0.05) between exercised and non-exercised legs are marked with an asterisk (*). Note the overall similarities in fiber area alterations between the exercised and non-exercised muscles. Furthemore, note also the more marked changes in fiber area and capillary supply among fibers containing MyHCII compared with fibers containing MyHCI. Bars indicate SD.

**Figure 10:**
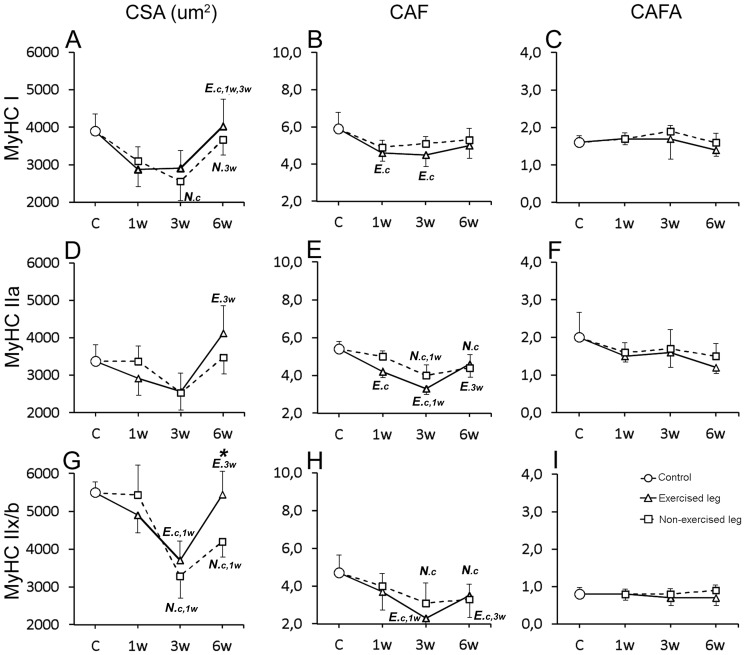
Graphs showing fiber area and capillary parameters for fiber types in the exercised and non-exercised gastrocnemius muscle after 1w, 3w and 6w of unilateral exercise. Figure show changes in; muscle fiber cross-sectional area (CSA) (A, D, G), number of capillaries around each individual fiber (CAF) (B, E, H), and number of capillaries around fibers relative to cross-sectional fiber area (CAFA) (C, F, I) for fibers containing MyHCI, MyHCIIa and MyHCIIx/b. The mean group values in the figure are connected with lines to facilitate interpretation of the direction of changes after E/EMS, but they do not imply that the changes between the sample points represent a linear change over this period. Exercised leg is defined by continuous black line, non-exercised leg by dotted line. Significant difference (p<0.05) within the exercised leg is marked with *E* and significant difference within the non-exercised leg is marked with *N*. Significant differences relative to controls are marked (c), to 1w group (1w) and to 3w group (3w). Significant differences (p<0.05) between exercised and non-exercised sides are marked with an asterisk (*). Note the overall similarities in changes for CSA and CAF for all fiber types between the exercised and non-exercised muscles. Bars indicate SD.

In comparison to controls, alterations in frequency of fiber phenotypes, fiber size and capillary supply were noted for the experimental animals in both the exercised and non-exercised soleus and gastrocnemius muscles after E/EMS (Figs. 1, 4–6). A summarized overview of the changes is given below. Details for all parameters and statistical differences within and between exercised and non-exercised legs at 1w, 3w and 6w and differences to controls are given in Figs. 7–10.

### The soleus muscle

#### Controls

The control animals had a muscle morphology characterized by densely packed polygonal fibers of about similar sizes. The muscle was mainly composed of MyHCI fibers (98.1%), a low proportion of MyHCIIa fibers (1.4%) and a few hybrid MyHCI+IIa (0.5%) fibers (Figs. 6 and 7). The mean fiber cross-sectional area (CSA) was 5183±1243 µm^2^, with no significant difference in size between the different fiber types. The mean number of capillaries around fibers (CAF) was 7.7. When CAF was related to area for each individual fiber (CAFA), the mean value was 1.6. The mean CAF and CAFA values were higher for MyHCI than MyHCII fibers (p<0.05). The number of capillaries per muscle area, i.e. the capillary density (CD), was in mean 462 cap/mm^2^ (Fig. 8).

#### Muscle fiber type composition

After 6w of exercise, the mean percentage of MyHCIIa fibers was higher and the mean the percentage of MyHCI fibers was lower in both exercised and non-exercised legs compared to controls, with significantly lower values for MyHCI fibers in the non-exercised side (88.1% vs. 98.1%, p<0.01) (Figs. 6 and 7). In addition, the percentage of hybrid fibers co-expressing MyHCI and MyHCIIa was in the non-exercised 6w group higher than in the controls (4.6% vs. 0.5%), while the exercised leg lacked this fiber type (Fig. 7).

#### Muscle fiber cross-sectional area (CSA)

After 3w of exercise, the mean fiber CSA was in both legs smaller than in controls (E 4420 µm^2^ and NE 4333 µm^2^, respectively vs. 5183 µm^2^, p<0.05). At 6w, the mean CSA of fibers in the exercised leg was higher than at 3w i.e. a fiber size in same range as in controls, while CSA in the non-exercised side still was lower than in controls (4153 vs. 5183 µm^2^, respectively, p = 0.08) (Fig. 8). Changes in fiber size were found for both MyHCI and MyHCIIa fiber types (Fig. 9).

#### Capillary supply of muscle fibers

The number of capillaries around each individual fiber (CAF) was in the exercised leg lower after 3w of exercise than in controls (6.0 vs. 7.7, respectively, p<0.02), while CAF in the non-exercised leg was unchanged. After 6w CAF was in both legs lower than in controls (E 6.7 and NE 6.5, respectively vs. 7.7). The drop in CAF in the non-exercised leg at 6w was significant against the 1w and 3w groups (p<0.04). Comparison between both legs showed significantly lower CAF values in the exercised than non-exercised side at 1w (p<0.05) and 3w (p<0.01), but not at 6w (Fig. 8). When relating CAF to fiber area, the CAFA value was significantly higher in the non-exercised leg at 3w compared to controls (2.0 vs. 1.6, p<0.01). The high CAFA value after 3w of exercise relates to the smaller fiber size but unchanged CAF (Fig. 8). When comparing CAFA within between both legs, the values were significantly higher in the non-exercised than in the exercised leg at both 3w (p = 0.001) and 6w (p<0.05) (Fig. 8).

#### Capillary density (CD)

In the exercised leg, there was no significant changes in CD, while in the non-exercised side, CD was significantly higher at 3w and 6w as compared to both control and 1w groups (p<0.04). Comparison between the two legs showed significantly higher CD in the non-exercised side than in the exercised side after 3w (p<0.01) and 6w of E/EMS (p<0.01) (Fig. 8).

### The gastrocnemius muscle

#### Controls

The control muscles had normal fiber morphology with densely packed polygonal fibers. The muscle was composed of MyHCI (20.9%), MyHCIIa (15.8%), MyHCIIx/b (55.6%) and hybrid MyHCI+IIa (3.5%) and MyHCIIa+IIx/b (4.1%) fibers (Figs. 6 and 7). The mean value for fiber CSA was 4697 µm^2^, MyHCIIx fibers having a larger CSA (5494 µm^2^) compared to the other fiber types (ranged 3278 to 4008 µm^2^). The mean CAF and CAFA values were 5.2 and 1.1, respectively, and the mean CD was 326 cap/mm^2^. MyHCI fibers had the highest mean CAF and CAFA values, whereas fibers containing MyHCIIx/b had the lowest value (Fig. 10).

#### Muscle fiber type composition

After exercise, the mean percentage of MyHCI fibers was in both the exercised and non-exercised legs lower than in controls and the mean percentage of MyHCIIx/b fibers was, with one exception, higher. The difference was in the exercised leg significant for MyHCI fibers after 3w and 6w of E/EMS (9.5% and 10.1%, respectively vs. 20.9%, p<0.01). In the non-exercised leg, the percentage of MyHCIIa fibers was significantly higher at 6w than in all other groups (25.7% vs. 12.4 to 15.8%, p<0.03) (Fig. 7). Comparison between the two legs showed a lower frequency of MyHCI fibers in the exercised leg at 3w and 6w (p<0.05), and higher frequency of MyHCIIa fibers in the non-exercised leg at 6w (p<0.02) (Figs. 6 and 7).

#### Muscle fiber cross-sectional area (CSA)

After 3w of exercise, the fiber CSA was in both legs smaller than in controls (E 3572 µm2 and NE 3572 µm^2^, respectively vs. 4697 µm^2^, p<0.02). Smaller fiber size was significant for MyHCIIx/b fibers in both legs (p<0.003) and for MyHCI fibers in the non-exercised leg (p<0.02) (Fig. 10). After 6w of exercise, the fiber size of both legs was significantly larger than at 3w (p<0.05), with no significant differences to the control. Higher CSA values after 3w than 6w was found for MyHCI, MyHCIIa and MyHCIIx/b fibers in the exercised leg (p<0.05) and for MyHCI fibers in the non-exercised leg (p = 0.03). Comparison between the two legs showed significantly larger CSA values in the exercised than in the non-exercised side at 6w (4911 vs. 3940 µm^2^, p = 0.03) (Fig. 8).

#### Capillary supply of muscle fibers

After 3w and 6w of exercise, the mean CAF values of both exercised and non-exercised legs were significantly lower than in controls (CAF 2.7 to 4.0 vs. 5.2, p<0.05) (Fig. 8). In the exercised leg, significantly lower mean CAF values were found for MyHCI, MyHCIIa and MyHCIIx/b fibers at 3w and for MyHCIIx/b fibers at 6w (P<0.05) (Fig. 6). In the non-exercised leg, CAF was lower for MyHCIIa and MyHCIIx/b fibers at 3w and for MyHCIIx/b fibers at 6w (p<0.05) (Fig. 10). The CAFA values were in non-exercised leg higher at 3w than in controls and at 1w of exercise (1.4 vs. 1,1 respectively, p<0.04). The high CAFA value at 3w was correlated to a larger drop in fiber size but not in CAF. Comparison between the two legs showed significantly higher CAFA in the non-exercised leg at both 3w and 6w (p<0.03) (Fig. 8).

#### Capillary density (CD)

After 6w of E/EMS, CD in the exercised leg was lower as compared to controls (p<0.05). No significant difference in CD was observed in the non-exercised side. Comparison between the two legs showed significantly lower CD values in the exercised leg than in the non-exercised side at 6w (p<0.03) (Fig. 8).

## Discussion

The major finding of this study was that extensive unloaded exercise induced by EMS of one leg caused changes in fiber phenotype composition, fiber size and fiber vascularization not only in the exercised muscle, but also in the homologous non-exercised muscle in the contralateral leg. In addition, in restricted areas of the muscle tissue of both legs, the intervention caused inflammation, muscle fiber injury and axonal loss in nerves (c.f. [15]). In parallel to these changes, there were also signs of a process of tissue repair. Although the structural changes in both the manipulated and the resting leg mirrored each other, there were differences in the extent of the alterations. While changes in fiber type composition, fiber size and muscle capillarization were more pronounced in the fast phenotype gastrocnemius muscle, fiber injury, myositis, and fibrosis generally were more severe in the slow phenotype soleus muscle. Our findings provide support to previous suggestions that unilateral exercise can cause both adaptive and deleterious cross-transfer effects related to the locomotors system.

### Changes in muscle fiber composition

The findings that the experiment caused a shift from fibers expressing slow MyHCI in a direction towards fibers expressing fast MyHCII in both the soleus and gastrocnemius muscles is of specific interest. It is well established that increased neuromuscular activity and mechanical loading can induce a transition from faster to slower fiber phenotypes [22]. A Few reports have, however, shown evidence for fiber type transitions from slow MyHCI to fast MyHCII fibers after exercise [49]. Liu et al. [50] reported that long-term high velocity-training movements stimulated a shift from slow to fast fiber types. A similar finding was observed by Paddon-Jones et al. [51], who showed that fast isokenetic resistance training decreased the proportion of type I fibers (MyHCI) with a consistent increase in type IIb fibers (MyHCIIx). Furthermore, a bidirectional transformation of fibers from MyHCI and MyHCIIx/b to MyHCIIa has been reported after sprint training [52], [53] and all-out sprints on the cycle ergometer [54]. Interestingly, slow to fast transition has actually also been reported after long-term complete inactivity [55], spinal cord injury [56], microgravity [57] or experimentally by using phasic low or high frequency EMS [58], [59]. Since changes in neuromuscular activity secondary to exercise or EMS is considered to be a major cause of changes in muscle fiber phenotype composition [60], [61], the increased proportion of fibers containing fast MyHCII in the exercised leg probably relate to the experimental design with the use of unloaded high velocity exercise by EMS. However, the cause to the parallel shift in the non-exercised leg is unclear.

Although the bilateral shift in fiber type proportions mirrored each other, there were significant differences between the two muscles and between the exercised and non-exercised legs. In the slow soleus muscle, which contained 98% slow MyHCI fibers, the proportion of MyHCI fibers was decreased in the muscles of both legs after exercise whereas the proportion of fibers expressing MyHCIIa was increased. Moreover, while hybrid fibers co-expressing MyHCI and MyHCII were relatively common in the non-exercised leg, these fibers were rare in controls and non-existing in the exercised leg. These findings reflect a shift, especially in the non-exercised leg, towards a fiber phenotype profile better suited for fast contractions during aerobic conditions. In the gastrocnemius muscle, which contained 78% fast MyHC fibers, the bilateral shift was after 3w of exercise directed towards a higher proportion of fast fibers expressing MyHCIIx/b, the predominating MyHC isoform in the muscle. This trend was in the exercised leg sustained after 6w of exercise, while in the non-exercised leg there was transformation towards a higher proportion of fibers expressing MyHCIIa in the expense of fibers expressing MyHCIIx/b. In consideration of the changes in the SOL and GA muscles, unloaded exercise by E/EMS seems to cause bilateral changes in fiber type composition in the same direction, but the changes in the exercised legs may overtime also be influenced by muscle genotype.

The smaller fiber size (CSA) in the soleus and gastrocnemius muscles of both legs after 3w of exercise can be due to several causes. Firstly, in the exercised leg this type of overuse intervention may result in a higher level of degradation than synthesis of muscle proteins. The more pronounced changes in fiber size in the fast gastrocnemius than in the slow soleus muscle suggests that this type of intervention more affects a muscle predominated by fast MyHCIIx/b than slow MyHCI. Secondly, the bilateral signs of muscle fiber injury and axonal loss in certain nerves in the areas with myositis [15], [16] support denervation and fiber degeneration as one cause to loss of muscle fiber mass. Since muscles have the ability to go through a regenerative process after injury, newly formed fibers originating from myogenic stem cells, i.e activated satellite cells, might also contribute to the smaller mean fiber size values [62]. The observation of a high number of small-sized fibers expressing embryonic MyHC in the muscles of both exercised and non-exercised legs, but not in the controls, support fiber regeneration as one cause for the smaller mean fiber size. Embryonic MyHC is normally a useful marker for early stages of muscle fibre development. When the fibers develop and maturate, this MyHC isoform usually disappear and are replaced by adult slow or fast MyHC isoforms [24]. The greater variability and larger size of the fibers expressing embryonic MyHC in the exercised than in the non-exercised side indicate that the degenerative/regenerative process occurred at an earlier stage in the exercised side. The return to a more normal size of the fiber population in the exercised muscles after 6w of E/MS, may be due to the fact that regenerating fibers had increased in size and/or that the degenerative process diminished because muscle fibers have adapted to the intense strength regimen with a more balanced metabolism and protein synthesis.

### Changes in muscle vascularization

The findings that the unilateral intervention caused a significant regression of capillaries around muscle fibers in both legs are of specific interest. In the soleus muscle, and especially in the non-exercised leg, the loss of capillaries was observed in a later stage and at a lower magnitude than in the gastrocnemius muscle. The modification in number of capillaries per fiber was generally closely associated with the changes in fiber area in every experimental groups. One exception was observed for the non-exercised soleus muscle, where the capillary network was more or less unchanged after 1w and 3w of E/EMS, despite significantly smaller fiber size. This mismatch, as evidenced by the increased CAFA value, indicates a higher loss of muscle fiber mass than degradation of capillaries during the first weeks of experiment. However, after 6w of E/EMS, the capillary network had regressed and the capillary supply of fibers was now well correlated to the smaller fiber size also in the non-exercised soleus muscle.

It is unclear whether the bilateral changes in the microvascular system is an adaptive process accompanying the changes in the fiber size or if capillary damages also contributes to a degenerative process in the muscle. Previous studies have shown that strenuous exercise [63], [64], EMS [65] and inflammation can damage the capillary endothelial cells and cause capillary degradation [66]. Any change in the extent of the microcirculation risks to upset the balance between blood support and metabolic requirements. Thus, a limitation of blood supply in combination with an exaggerated physical activity may over time cause muscle fiber damage [67]. It is also possible that the use of EMS affects the blood flow to the muscle tissue by having effects on the contracting smooth muscle layer in supporting vessels. A disturbed auto-regulation of the blood flow might have negative consequences for the muscle tissue and it is possible that cross-transfer effects can contribute to disturbed blood flow also in the contralateral side. Interestingly, Hudlicka et al. [65] demonstrated that bilateral changes in the ultrastructure of the capillary walls and capillary swelling after unilateral chronic low frequency EMS was caused by both increased muscle activity and by orthodromic activation of afferent nerve fibers. Elimination of afferent input by section of the dorsal roots attenuated proliferation and capillary swelling in the ipsilateral muscles and eliminated them on the contralateral side. The orthodromic activation of afferent nerve fibers was supposed to result in release of signal substances as neuropeptide tachykinin substance P (SP) that lead to the changes in both stimulated and contralateral non-stimulated muscles. Our previous finding of a bilateral up-regulation of SP and its preferred receptor neurokinin1 (NK1-R) indeed suggests involvement of the tachykinin system in the bilateral inflammatory degenerative process [15], [16]. Evidence of bilateral up-regulation of SP and NK-1R has been reported after unilateral retinal laser burn-induced neuropathy [68] and unilateral burn injury in one limb [69].

Besides the occurrence of muscle fiber alterations and degradation of capillaries, there were also specific changes of the vascularization in the areas with myositis in both the exercised and non-exercised legs. Two interesting features should here be pointed out. Firstly, in the focal areas with severe inflammation there were an increased number of enlarged capillaries and vessels with a size similar to that of arterioles or venules. This observation implies that the inflammatory process dilates capillaries and triggers growth of arterioles/venules to increase blood flow to the area [70], [71]. Secondly, in adjacent areas with less inflammation but with an increased amount of connective tissue and fat infiltration, there were few vessels. Consequently, muscle fibers in these areas were generally supplied by a low numbers of capillaries indicating a low oxygenation of the tissue. Since an intact microvascular supply is required for an adequate regeneration [72], an inflammatory process damaging capillaries might affect the possibility for muscle regeneration. Thus, when the inflammatory process is followed by defective muscle fiber regeneration, perhaps as a consequence of detrimental capillary degradation, parts of the affected area will in the long run be replaced by fibrous connective tissue. Based on the seemingly destructive consequences of myositis, it is reasonable to believe that a part of the histopathological and adaptive changes in the adjacent areas of focal mysositis in both the exercised and non-exercised muscles can be considered to be a effect of inflammation and capillary destruction.

### Cross-transfer effects

Even if our findings strongly indicate on an involvement of the nervous system in the cross-transfer effects, it cannot be determined to what extent the contralateral changes is a consequence of cross-transfer effects of the nervous system or if other systems or mechanisms are involved. Electromyography (EMG) recordings have for example demonstrated some nervous activity in the contralateral muscles during unilateral exercise, however this effect seems to be rather small [73], [74] and no muscle activity were observed in the resting leg during the experiment. Hypothetically, the contralateral alterations could be related to muscle fatigue in the exercised leg, causing a compensatory neuromuscular activity in the non-exercised leg in order to maintain posture during movements in-between the E/EMS sessions. Against this proposal is the fact that we did not observe amended movements or changed behaviors in the rabbits in-between the experimental periods. In addition, such type of activity does not match with known patterns of fiber type transformation. The possibility that the bilateral changes in this study relate to changed levels of humoral substances having a modulating effect on the muscle motor system cannot be excluded [75]–[77]. However, the presence of myositis and histopathological changes only in focal areas of the muscles contradicts a circulatory effect as a major cause. Furthermore, it is known that ligating the draining venous system of the inflamed area prior to an insult does not abolish the contralateral response [78], while a lesion of nociceptive nerves supplying either the contralateral or the ipsilateral limb prior to inflammatory insults, abolishes the contralateral responses [21]. Nevertheless, the overall results of this study clearly points towards a signaling system across the midline of the body. The findings of symmetric changes in fiber morphology, muscle vascularization and development of bilateral myositis after E/EMS provide further support that manipulation or injury in one side of the locomotor system can cause cross-transfer effects to the contralateral side [17], [65].

It is believed that the increased strength in the contralateral limb after unilateral exercise is related to a process of motor learning of the brain rather than changes in the muscle morphology, i.e motor areas in the brain that are responsible for motor control have adapted to unilateral voluntary training and the opposite hemisphere in the brain may have access to these modifications [3], [4]. Unilateral training have also been proposed to enhance the organization of the spinal and cortical motor pathways to the contralateral limb, leading to an increased drive to the untrained limb [4]. However, the morphological changes and deleterious cross-transfer effects seen in this study may be caused by still another neuronal mechanism. There is thus some evidence of a commissural system in the spinal cord that can mediate transmedian signaling through inter-neurons to the contralateral side with a fairly precise bilateral presentation [17]. Since EMS bypasses motor activity through the central nervous system, it seem far-fetched to suggest that the exercise-induced overuse leads to sensory and antidromic nerve activity that pass over at the spinal level to the opposite side and secondarily lead to contralateral consequences. The existence of a commissural system might explain our and others observations of a cross-transfer up-regulation of neuropeptides in the contralateral side after unilateral experiments [79]. The precise background for the cross-over effect by the use of our model should nevertheless await further studies.

Regardless of the cause, our study shows that the experimental model with frequent unilateral unloaded exercise by EMS causes contralateral effects in muscles. Since cross-transfer effects may be both harmful and of positive nature, the occurrence of contralateral cross-transfer processes should be considered in clinical situations with immobilization/exercise of only one limb and in research concerning the symmetric spreading of inflammatory disease or pain.

### Conclusion

This study provides new evidence of both adaptive and degenerative cross-transfer effects across the midline of the body after unilateral manipulation of one of the extremities. The results show that excessive repetitive unilateral exercise by EMS causes not only fiber injury and myositis in focal areas of the muscle, but also bilateral changes in fiber phenotype composition, fiber size and vascularization. Although both the soleus and gastrocnemius muscles were affected by the experiment, the bilateral reactions in the two muscles were not identical, indicating that the muscle phenotype has an impact on the consequences of the manipulation. The results from this study may not be directly transferable to the human situation, yet the findings can be important to consider in a wide range of musculoskeletal and neuromuscular disorders. It is also important to consider possible cross-over effects when the contralateral muscle is used as a control, and especially when EMS is the driving force of muscle contractions.
